# Ascertaining the optimal myoelectric signal recording duration for pattern recognition based prostheses control

**DOI:** 10.3389/fnins.2023.1018037

**Published:** 2023-02-22

**Authors:** Mojisola Grace Asogbon, Oluwarotimi Williams Samuel, Ejay Nsugbe, Yongcheng Li, Frank Kulwa, Deogratias Mzurikwao, Shixiong Chen, Guanglin Li

**Affiliations:** ^1^CAS Key Laboratory of Human-Machine Intelligence-Synergy Systems, Shenzhen Institutes of Advanced Technology (SIAT), Chinese Academy of Sciences (CAS), Shenzhen Institute of Artificial Intelligence and Robotics for Society, Shenzhen, China; ^2^Nsugbe Research Labs, Swindon, United Kingdom; ^3^Unit of Biomedical Engineering, Department of Physiology, School of Engineering, Muhimbili University of Health and Allied Sciences, Dar es Salaam, Tanzania

**Keywords:** electromyogram (EMG), finger gestures, pattern recognition (PR), prostheses, signal recording duration (SRD)

## Abstract

**Introduction:**

Electromyogram-based pattern recognition (EMG-PR) has been widely considered an essentially intuitive control method for multifunctional upper limb prostheses. A crucial aspect of the scheme is the EMG signal recording duration (SRD) from which requisite motor tasks are characterized per time, impacting the system’s overall performance. For instance, lengthy SRD inevitably introduces fatigue (that alters the muscle contraction patterns of specific limb motions) and may incur high computational costs in building the motion intent decoder, resulting in inadequate prosthetic control and controller delay in practical usage. Conversely, relatively shorter SRD may lead to reduced data collection durations that, among other advantages, allow for more convenient prosthesis recalibration protocols. Therefore, determining the optimal SRD required to characterize limb motion intents adequately that will aid intuitive PR-based control remains an open research question.

**Method:**

This study systematically investigated the impact and generalizability of varying lengths of myoelectric SRD on the characterization of multiple classes of finger gestures. The investigation involved characterizing fifteen classes of finger gestures performed by eight normally limb subjects using various groups of EMG SRD including 1, 5, 10, 15, and 20 s. Two different training strategies including Between SRD and Within-SRD were implemented across three popular machine learning classifiers and three time-domain features to investigate the impact of SRD on EMG-PR motion intent decoder.

**Result:**

The between-SRD strategy results which is a reflection of the practical scenario showed that an SRD greater than 5 s but less than or equal to 10 s (>5 and < = 10 s) would be required to achieve decent average finger gesture decoding accuracy for all feature-classifier combinations. Notably, lengthier SRD would incur more acquisition and implementation time and vice-versa. In inclusion, the study’s findings provide insight and guidance into selecting appropriate SRD that would aid inadequate characterization of multiple classes of limb motion tasks in PR-based control schemes for multifunctional prostheses.

## 1. Introduction

Upper limb loss precludes amputees from full exploration of their environment especially in accomplishing tasks that require their arm functions (Cordella et al., 2016; Wheaton, 2017). The varied setbacks faced by amputees during daily life activities have spurred the development of intelligent prosthetic limbs meant to intuitively restore their limb functions. At the forefront of this technology are myoelectric pattern recognition (PR) based prostheses that use decoded motion intent from surface electromyogram (EMG) signals for their control (Cordella et al., 2016; Vujaklija et al., 2016; Parajuli et al., 2019). In an archetypal PR-based prosthetic control pipeline, EMG signals of coordinated muscle activities of specific limb motion are recorded, processed, and motor tasks are decoded *via* machine learning algorithms which serve as control inputs to the device (Li et al., 2010; Asogbon et al., 2020a; Nsugbe et al., 2021a).

A number of confounding factors that impede the clinical and commercial relevance of PR-based prostheses in practical settings have been well studied with solutions proposed in recent years (Fougner et al., 2010; Lorrain et al., 2010; Tkach et al., 2010; Young et al., 2011; He et al., 2013; Qing et al., 2021). For instance, electrode shift (Young et al., 2011), muscle contraction force variation (Lorrain et al., 2010; Tkach et al., 2010), abrupt alteration in limb position (Fougner et al., 2010), and variability arising from long-term EMG recordings (He et al., 2013), etc., are confounding factors that have been researched with potential solutions proposed. Despite these advances, an essential aspect that the above factors and many others rely upon is the EMG signal recording duration (SRD) per time that may impact the characterization of finger gestures. To the best of the author’s knowledge, EMG SRD has not been studied to date. For instance, when the EMG SRD is relatively lengthy, phenomena such as muscle fatigue is inevitable and may alter the muscle contraction patterns of specific limb motions; which may undermine the decoding of finger movements and, by extension, degrades the prosthesis control performance. In addition, long SRD often leads to relatively larger volume features, and classifier training time and may result in computational complexity and increased controller delay in real-time usage. On the other hand, somewhat shorter SRD may lead to reduced data collection durations that, among other advantages, allow for more convenient prosthesis recalibration protocols. On the other hand, signals acquired using short SRD may result in poor motion gesture recognition if sufficient/adequate neural information is not contained in the signal, especially if it is collected from amputee patients.

To date, several existing studies have arbitrarily employed varied myoelectric SRD for decoding targeted limb motions in the context of PR-based prostheses without taking into consideration whether or not they would yield optimal characterization of the motor tasks. For instance, Cengiz and Demir (2020) acquired myoelectric and gyroscopic signals of multi-class finger gestures with SRD of 5 s and Al-Timemy et al. (2015) investigated the influence of muscle contraction force variation on the classification performance of the EMG-PR system using SRD in the range of 8–12 s. In addition, Li et al. (2021) used an SRD of 4 s in a study aimed at enhancing the motion classification accuracy of the EMG-PR system and Samuel et al. (2018b) utilized an SRD of 5 s in a study aimed at improving the EMG-based features for prostheses control. While we acknowledge that several efforts have been made toward tackling pertinent issues in the field of PR-based prostheses technology (Cordella et al., 2016; Bates et al., 2020; Nsugbe et al., 2021b), the investigation of the optimal myoelectric SRD remains an open research question. Hence, it is essentially necessary to investigate and determine the optimal myoelectric SRD that would aid adequate characterization of amputees’ limb motion intents and by extension the intuitive control of multifunctional prostheses. Also, the investigation should provide researchers and developers in the field with proper insight and guidance on the selection of appropriate SRD when conducting research or developing a requisite solution.

Therefore, this study systematically examined the impact of varied lengths of myoelectric SRD on the characterization of motor intents associated with multiple classes of fine-finger gestures performed by recruited subjects. More specifically, the experiments involved eight normally limb subjects (including six males and two females with no muscular or neurological disorder history), and each subject elicited fifteen classes (single and combined classes) of finger gestures under varying durations of EMG-SRD including 1, 5, 10, 15, and 20 s. Afterward, each SRD was pre-processed, notable feature extraction methods were applied for feature vector construction, and the feature vector was employed to build three distinct machine learning classification algorithms for the decoding of the finger gestures based on Within-SRD and Between-SRD strategies, which are described in the methodology section. Benchmark performance metrics were applied to evaluate the gesture pattern characterization and their corresponding decoding performance for each SRD.

## 2. Materials and methods

### 2.1. Data collection and processing

**FIGURE 1:**
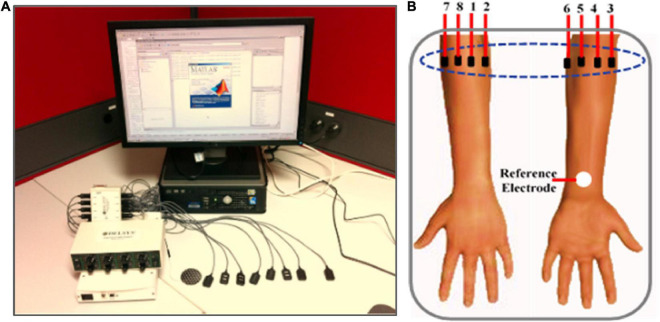
**(A)** EMG data acquisition system (Delsys Inc.) setup; **(B)** electrodes placements on the anterior and posterior on the participant’s right arm (Khushaba and Kodagoda, 2012).

The myoelectric dataset utilized in this study was acquired from an online EMG datasets repository (OneDrive). The signal was collected using BagnoliTM EMG Acquisition System (manufactured by Delsys Inc.). The equipment setup and electrode placement scheme is shown in Figures 1A, B. Prior to the data collection process, a total of eight normally limb subjects including six males and two females with no history of muscular or neurological disorders were recruited and informed about the study’s objectives (Khushaba and Kodagoda, 2012). Before their inclusion in the experiment, written informed consent was obtained from each subject, indicating their willingness to participate in the study. Afterward, eight EMG signal sensors were placed over the forearm muscles of each subject and a dual-slot adhesive skin interface was applied to firmly fix the electrodes to the skin to prevent undesirable displacement that may affect the quality of the signals. Besides, a reference electrode was placed on the wrist of each of the participants as shown in Figure 1B.

For the data recording task, the participants were instructed to sit down on a chair in a comfortable manner with their arms supported and fixed at a specific position (to ensure consistent arm position throughout the experiment). And fifteen classes of finger gestures were elicited as shown in Table 1 where each motion class lasted for a period of 20 s and was followed by a rest period of 5 s. Meanwhile, EMG recordings of three experimental trials were utilized in this study (Khushaba and Kodagoda, 2012).

**TABLE 1:** Fifteen classes of finger motions with their respective codes.

Motion group	SN	Motion classes	Code
Flexion of each individual fingers	1	Thumb	T
	2	Index	I
	3	Middle	M
	4	Ring	R
	5	Little	L
Combined fingers motions	6	Thumb-index	TI
	7	Thumb-middle	TM
	8	Thumb-ring	TR
	9	Thumb-little	TL
	10	Hand close	HC
	11	Index-middle	IM
	12	Middle-ring	MR
	13	Ring-little	RL
	14	Index-middle-ring	IMR
	15	Middle-ring-little	MRL

The recorded EMG signals were amplified using a Delsys Bagnoli-8 amplifier to a total gain of 1000 while the signal was sampled at the rate of 4000 Hz. A bandpass filter between 20 and 450 Hz and a notch filter was applied to the signal to process and eliminate power line interference.

### 2.2. Feature extraction

**FIGURE 2:**
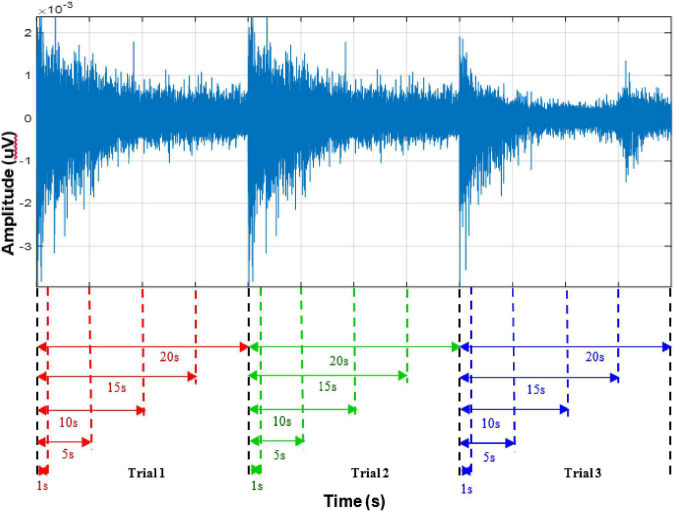
Conceptualized diagram of varying signal recording lengths considered in the study for three trials.

To investigate the impact of SRD on the characterization of the multiple classes of figure motions, different lengths of EMG signal recordings (1, 5, 10, 15, and 20 s), as conceptualized in Figure 2 were examined. Each SRD data was analyzed by partitioning each motion duration into a series of analysis windows with a length of 150 and 100 ms increments *via* an overlapping segmentation scheme, which has been commonly applied in the field of EMG signals processing (Englehart and Hudgins, 2003; Menon et al., 2011; Asogbon et al., 2020b). The segmentation process is often carried out to enhance the performance and response time of the PR-based myoelectric control scheme in practical settings (Asogbon et al., 2020b).

From each analysis window segment of the EMG signal, three different features whose mathematical expressions are presented in (Eqs 1–3) were extracted individually to build a machine learning classifier for decoding the different classes of finger motions. It should be noted that the feature extraction methods have been widely applied for characterizing multiple classes of targeted limb motions and they include the Hudgins’ time-domain feature set (mean absolute value: MAV, number of zero crossings: ZC, waveform length: WL, and number of slope sign changes: SSC), Novel Time-Domain Feature (NTDF, proposed by our research team), and the Root Mean Square (RMS) (Hudgins et al., 1993; Englehart and Hudgins, 2003; Samuel et al., 2018a; Asogbon et al., 2020a,b).


$$M\,A\,V=\frac{1}{\text{k}}\,\sum_{n=1}^{\text{k}}\left|{\text{x}}_{\text{n}}\right|$$

(1) $$\text{WL}=\sum_{n=1}^{k-1}\left[\left(\left|{\text{x}}_{n+1}-{\text{x}}_{\text{n}}\right|\right)\right]$$

$$\text{ZC}=\sum_{n=2}^{k-1}\left[\left({\text{x}}_{\text{n}}-{\text{x}}_{n-1}\right)*\left({\text{x}}_{\text{n}}-{\text{x}}_{n+1}\right)\right]$$

$$SSC=\sum_{n=1}^{k-1}\left[\text{sgn}\left({\text{x}}_{\text{n}}*{\text{x}}_{n+1}\right)\cap \left({\text{x}}_{\text{n}}-{\text{x}}_{n+1}\right)\geq \mathrm{Thr}.\right]$$

Where *x_n_*is the *n^th^* sample in a given segment of the EMG recordings of length *k*. Briefly, the *MAV* represents an estimate of the mean absolute value of *x* in a given segment of the signal which is of length *k, WL* provides information regarding the wavelength characteristics in a given segment of the signal, *ZC* represents a frequency measure of the number of times the waveform crosses zero (baseline), and *SSC* denotes an alternative but complementary measure of the number of times the slope changes sign (Hudgins et al., 1993; Englehart and Hudgins, 2003). And an aggregation of these descriptors forms the TD4 feature set that was adopted in the subsequent section of the manuscript (Hudgins et al., 1993; Englehart and Hudgins, 2003). Meanwhile, the *Thr. (with a value of 0.01)* represent the threshold upon which the *SCC* value is computed.


$$S\,I\,S=\sum_{n=0}^{N-1}x\,{\left[n\right]}^{2}$$

$$normRSD1=\;\frac{1}{N}\sum_{n=0}^{N-1}dx_{1}{\left[n\right]}^{2}$$

$$n\,o\,r\,m\,R\,S\,D\,2=\frac{1}{N}\,\sum_{n=0}^{N-1}d\,x_{2}\,{\left[n\right]}^{2}$$

(2) $$normLogDet.=norm\left(e^{\frac{1}{N}\,\sum_{n=0}^{N-1}\text{log}\,\left(x\,\left[n\right]\right)}\right)$$

$$m\,M\,S\,R=\frac{1}{\text{k}}\,\sum_{n=1}^{\text{k}}\;{\left(x_{n}\right)}^{1/2}$$

$$m\,A\,S\,M=\left|\frac{\sum_{n=1}^{\text{k}}\;{\left(x_{n}\right)}^{e\,x\,p}}{k}\right|$$

$$exp\;=\;{\{}_{0.75,\;\;\;\;\;otherwise}^{0.50,\;\;\;\;\;if\;(n\geq \;0.25\;*\;k\;\&\&\;n\;\leq \;0.75)}\;$$

Where *SIS*the denotes the simple integral square which captures the energy content in a segment of EMG signal (*x[n]/x_*n*_*) and *N* denote the total length of the signal in a segment, the *normRSD1* and *normRSD2* represent the normalized form of the first and second order of the root squared descriptors, which captures the spectral information in a given EMG signal segment, and the *mMSR* and *mASM* descriptors capture an estimate of the power of the signal per segment (Asogbon et al., 2020a). And an aggregation of these descriptors forms the NTDF feature set that was employed subsequently (Asogbon et al., 2020a).


(3) R⁢M⁢S=1k⁢∑n=1kxn2

Where *RMS* denote the square root of the average power of EMG recordings (*x*_*n*_) in a given segment of the signal whose length is denoted by *k*.

After each of the above-mentioned features has been extracted, three widely utilized machine learning classification algorithms with simple and intuitive structure, high accuracy, and fast computation characteristics including the Linear Discriminant Analysis (LDA), K-Nearest Neighbor (KNN), and Random Forest classifiers (Boughorbel et al., 2017; Asogbon et al., 2021) were applied to classify the motion classes for the considered SRD groups. Thus, we examined the impact of varied SRD on finger movement pattern characterization using a fixed set of features and machine learning classifiers using two approaches described below.

### 2.3. Data analysis and performance evaluation

The effect of SRD on EMG-PR motion intent decoding was systematically investigated based on two varied types of training and testing strategies, namely Within-SRD Group (which is represents the commonly adopted approach) and Between-SRD Approach described as follows:

(a). Within-SRD Group Scenario: In this approach, the EMG-PR scheme’s performance was investigated when the training and testing feature vectors were constructed from EMG recordings of the same SRD group. Specifically, in this approach, the requisite feature vector is constructed from the first two trials (Trial 1 + Trial 2) of the recordings (designated as the training set) while the corresponding test set feature vector is obtained from the third trial (Trial 3).
(b). Between-SRD Group Scenario: In this approach, the EMG-PR scheme’s performance was examined when the feature vector constructed from a specific EMG SRD group (say 1 s) is used for training the classifier while the feature vector obtained from all the SRD groups (1, 5, 10, 15, and 20 s) is used for testing the classifier’s decoding performance. In addition, it should be noted that the training set is constructed from all the trials while the test set is also obtained based on all the trials.

For each of the approaches described above, evaluation metrics including classification accuracy (CA) and Mathew Correlation Coefficient (MCC) were considered and their descriptions are given as follows. The CA, a commonly used evaluation metric that represents the number of correctly classified samples over the sum of all samples [Eq. (4)] was utilized. The MCC metric which has been widely applied in multiclass problems was also adopted for evaluation in the study [Eqs (5, 6)]. MCC is considered to be a highly informative metric for assessing classification tasks since it is considered to be a balanced ratio amongst the four confusion matrix parameters (false positives, true positives, true negatives, and false negatives) (Liarokapis et al., 2014; Asogbon et al., 2020a).


(4) $$C\,A=\frac{N\,u\,m\,b\,e\,r\,o\,f\,c\,o\,r\,r\,e\,c\,t\,l\,y\,c\,l\,a\,s\,s\,i\,f\,i\,e\,d\,s\,a\,m\,p\,l\,e\,s}{T\,o\,t\,a\,l\,n\,u\,m\,b\,e\,r\,o\,f\,t\,e\,s\,t\,i\,n\,g\,s\,a\,m\,p\,l\,e\,s}*100\%$$

(5) $${\text{MCC}}_{j}=\frac{\left(T\,P*T\,N\right)-\left(F\,P*F\,N\right)}{\sqrt{\left(T\,P+F\,P\right)\,\left(T\,P+F\,N\right)\,\left(T\,N+F\,P\right)\,\left(T\,N+F\,N\right)}}$$

(6) $${\text{MCC}}_{a\,v\,e}=\frac{\sum_{j=1}^{n}{\text{MCC}}_{j}}{n\,c\,l\,a\,s\,s}$$

Where j = 1,2,3……number of classes (n, nclass), TP is the count of true positives, TN represents the count of true negatives, FP is the number of false positives, and FN is the number of false negatives as obtained from a confusion matrix. Meanwhile, the MCC value was computed using the macro-averaging technique.

Furthermore, the statistical significance test between the SRD groups was performed using the Friedman test with a confidence level set to *p* < 0.05. The statistical analysis was carried out in MATLAB.

## 3. Results

### 3.1. Within-SRD group scenario

#### 3.1.1. Performance evaluation of varying signal length on EMG-PR classifier across finger gesture tasks

**FIGURE 3:**
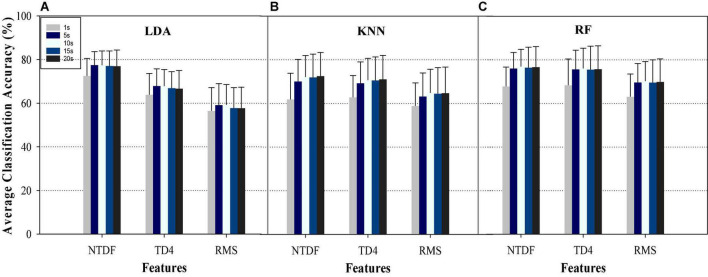
Average classification accuracies of the different groups of signal recording length based on the Within-SRD Group Scenario using **(A)** LDA, **(B)** KNN, and **(C)** RF for NTDF, TD4, and RMS features across finger gestures and participants.

Utilizing different classifiers and feature sets, Figure 3 presents the average classification accuracies across the fifteen-finger motion tasks across eight subjects. The aim here is to examine the impact of individual SRD (1, 5, 10, 15, and 20 s) on the classification performance EMG-PR system. It can be observed from the result presented in Figure 3 that the average classification accuracy varies across the features and classifiers. For instance, 5 s SRD achieved the highest CAs across subjects and finger gestures with an increment ranging from 0.01 to 3.86% for the LDA classifier when compared with the performance of the other SRD groups. Similarly, some worth different phenomenon was observed when KNN and RF classifiers were employed. The 10 s (for RF) and 20 s (for KNN) outperformed other SRD groups with an increment in the classification accuracies ranging from 0.22 to 8.26% and 0.33 to 7.99%, though with an insignificant difference with the other SRD groups except for 1 s SRD. In terms of feature performance, the NTDF achieved the highest average accuracies while RMS has the lowest accuracies for all the classifiers.

**FIGURE 4:**
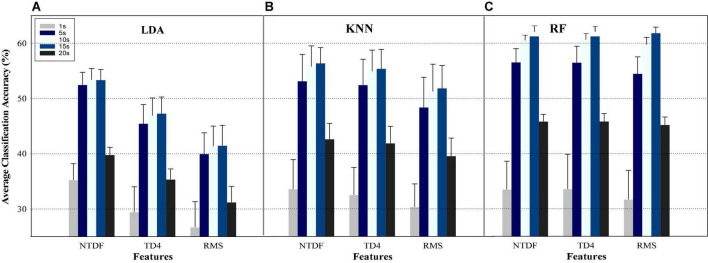
Average classification performance across finger gestures and participants based on Between-SRD Approach using the NTDF, TD4, and RMS for **(A)** LDA, **(B)** KNN, and **(C)** RF when each of the SRD was used as a training data and tested with all SRD dataset.

Overall, the combination of LDA + NTDF recorded the highest average classification accuracy rate compared to KNN, RF, and other features. Specifically, the 1 s SRD can be observed to be the least accuracy while the other SRDs achieved higher but similar accuracies compared to 1 s SRD for the LDA-NTDF combination. Analyzing the effect of the signal recording length based on classification accuracies reported in Figure 4 for all the classifiers and features, it can be seen that the classification accuracy of 1 s SRD is significantly (*p* > 0.05) lower than the other SRDs while 5 s has the highest accuracy though with almost the same decoding performance with 10, 15 and 20 s SRD (*p* > 0.05). It is worth noting that in this study, an increase in SRD incurs increased training and testing time, which may introduce some sort of delay in the performance of the prostheses in practical deployment. In other words, the longer the recording duration the higher the computational cost and vice-versa. One possible reason for the poor performance of the 1 s SRD could be due to a lack of adequate neural information in the signal length. This result indicates that an appropriate selection of crucial parameters/methods (such as signal recording length and feature-classifier combination) would greatly impact the overall performance of the EMG-PR-based motion intent decoding strategy employed in the control of multifunctional prostheses. Across all the classifiers, statistical analyses *via* Friedman’s test show no significant difference in decoding accuracies for the SRD groups for NTDF: *p* = 0.11 and TD4: *p* = 0.10, though with substantial increment between 1 s and the other SRDs. Meanwhile, there is a significant difference for RMS: *p* = 0.043.

### 3.2. Between-SRD group scenario

#### 3.2.1. Effect of signal recording duration on EMG-PR classification performance

The Within-SRD Scenario has been used in many existing works; however, it may be difficult to utilize this approach to select the optimal SRD for motion intent characterization because it is not practicable in real-life situations. Hence to determine the optimal SRD, we employed the Between-SRD Approach to systematically investigate the generalizability of each SRD group for movement intent decoding. In this scenario, the machine learning classifiers were trained with data concatenated across all the trials for a specific SRD (say, 1 s) and tested using data from all trials of all the SRD groups (1, 5, 10, 15, and 20 s) and the obtained results is shown in Figures 4A–C. This Figure depicts the average CA across the 15 classes of finger gesture and participants, and it could be seen that the CA decreased for all SRDs, features, and classifiers compared to the result presented for within-SRD scenario in Figure 3 (where the training and testing data are from the same SRD). Besides, for the Between-SRD scenario, the highest decoding performance was achieved by 15 s, followed by 10 s SRD (though with insignificant difference), while 1 and 20 s yielded the lowest CA for all features-classifier combinations. Examining the classifiers performance further, RF (Figure 4C) outperformed LDA (Figure 4A) and KNN (Figure 4B) for all the SRDs and features except for 1 s SRD for the combination of LDA + NTDF. For instance, for the most performing feature (NTDF) across motion classes and participants, RF achieved an increment of up to 7.09 and 4.71% compared to LDA and KNN, respectively, for 10 s SRD. In a similar manner, RF achieve an increment of 8.82 and 5.79% compared to LDA and KNN, correspondingly for 15 s SRD. Besides, across classifiers, the NTDF feature outperformed the other features for all the SRDs. Amid the classifiers, there are statistical significances among the SRDs for NTDF (*p* = 0.021), TD4 (*p* = 0.017), and RMS (*p* = 0.017). Similarly, significant differences occurred among the SRDs across features. From the statistical significance result, 10 and 15 s SRD achieved similar performance with no substantial difference when compared with each other. Furthermore, performance comparison between the results reported in Figure 4 for each SRD reveals that the Between-SRD Approach would significantly influence the decoding performance of the EMG-PR system.

It is worth mentioning that observations during the EMG-PR scheme’s implementation revealed that the computational cost generally increase with an increase in SRD, and this may necessitate us to consider an SRD that is greater than 5 s but less than or equal to 10 s (>5 s and < = 10 s).

#### 3.2.2. Evaluating signal duration effect based on MCC metric

The effect of the different groups of SRD on the characterization of the motor intent for the Between-SRD strategy was further examined using the MCC metric defined in section “2.3. Data analysis and performance evaluation” of the paper. Notably, the MCC is a highly informative evaluation method for estimating classification tasks mainly due to its ability to balance the ratio among the four confusion matrix parameters effectively. Thus, the corresponding MCC values for each group of signal length were computed from their respective confusion matrices.

**FIGURE 5:**
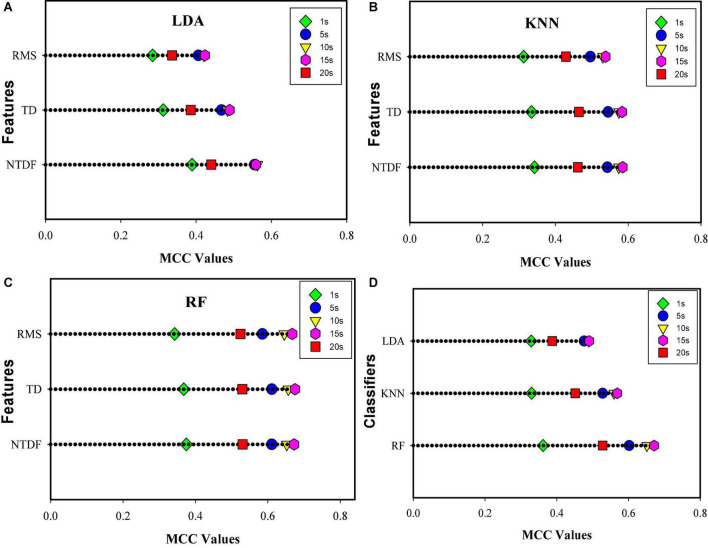
Average MCC values of the motion duration groups across finger gestures and participants based on Between-SRD Strategy using NTDF, TD4, and RMS for **(A)** LDA, **(B)** KNN, and **(C)** RF, **(D)** average MCC value using all the features, for LDA, KNN, and RF classifiers.

Using the same number of classifiers and features, the obtained average MCC value across all the finger motion tasks and subjects is shown in Figures 5A–D using horizontal dot plots. Inspecting each of the classifier-feature combinations (Figures 5A–C) for all the SRD groups, NTDF yielded the highest MCC values for LDA and similar values with TD4 for KNN and RF. Meanwhile, the RMS feature has the lowest MCC values for the different groups of signal recording lengths investigated.

Observing the performance of the SRDs from Figures 5A–C, an overlap of symbols could be seen for 5, 10, and 15 s for LDA, and 10 and 15 s symbols overlap for KNN and RF, respectively, indicating similar MCC values. For all the classifiers and features, 1 s achieved the lowest MCC value, followed by 20 s, while 10 and 15 s SRD obtained the highest MCC values, indicating consistency with the CA results described in section “3.2.1. Decoding performance for between-SRD scenario.” Compared to other classifiers, RF recorded the best values for RMS, TD4, and NTDF features. A similar performance trend of the classifiers and features for the SRD groups could be observed in the result presented in Figures 4A–C (see section “3.2.1. Decoding performance for between-SRD scenario”). And Figure 5D depicts classifier-wise computation of the mean MCC values across all features, motion tasks, and subjects. It can be seen that the RF classifier achieved the best MCC values of 0.36, 0.60, 0.65, 0.67, and 0.52 for 1, 5, 10, 15, and 20 s signal recording lengths, respectively. Furthermore, statistical analyses *via* Friedman’s test show a significant difference (*p* = 0.007) in decoding accuracies across SRDs and features for LDA, KNN, and RF classifiers. Also, statistical significance occurs between the SRD groups (0.017) across classifiers.

#### 3.2.3. Effect of signal recording length on individual finger gesture decoding

**FIGURE 6:**
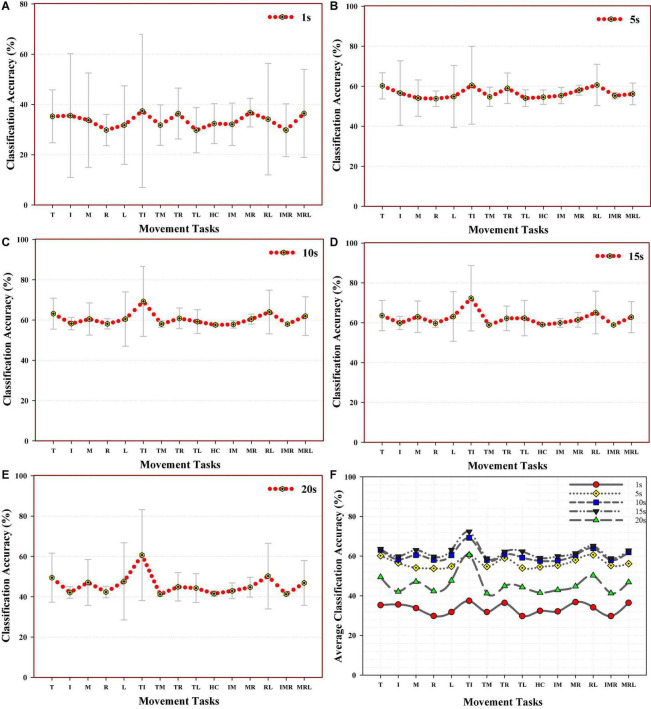
Average decoding performance across subjects for individual finger gesture using the combination of NTDF feature and LDA classifier for **(A)** 1 s, **(B)** 5 s, **(C)** 10 s, **(D)** 15 s, and **(E)** 20 s, **(F)** multiple splines curve lines and scatter plot showing performance comparison among the motion classes.

In this section, the recognition rate of each class of finger motion for individual signal recording length was examined across subjects using the combination of the NTDF feature and the RF classifier based on their performance in the Between-SRD. Utilizing line and scatter plots with error bars, the obtained result is shown in Figures 6A–F. It should be noted that the standard deviation across the subjects is shown with error bars in the plot. From the results, it can be seen that there are variations in the error bars for all finger gestures across the SRDs (1, 5, 10, 15, and 20 s). Specifically, in all the SRD groups, the thumb-index class (denoted by TI) recorded the highest decoding accuracies of 37.44, 60.51, 69.28, 72.36, and 60.68% for 1, 5, 10, 15, and 20 s, respectively. Also, for all the SRDs, this gesture (TI) has the highest standard error compared to other finger gestures, signifying performance variation across the participants.

On the other hand, the thumb-little (TL) finger gesture recorded the least performance for 1 s, class ring (I) for 5 s, hand close (HC) for 10 s, and thumb middle (TM) for 15 and 20 s SRD.

Comprehensively, the performance comparison between the groups revealed that the 15 s SRD group achieved the highest average recognition followed by the 10 s SRD group across all the classes of finger gestures compared to other SRDs. Meanwhile, for a clear comparison of the characterization of the motor intent across the groups, Figure 6F depicts the results obtained for all the groups of signal recording length. Careful analyses revealed that most classes achieved the best performance at 10 and 15 s signal recording length with most classes’ symbols overlapping with one another. This result reveals that some finger gestures’ decoding performance may depend on relatively longer SRDs while 1 s SRD can be seen to achieve the least decoding performance followed by 20 s SRD.

## 4. Discussion

Pattern recognition (PR)-based myoelectric system has been widely studied primarily because of its capability to provide control schemes that could aid seamless realization of multiple degrees of freedom functions in upper limb prosthetic technology (Cordella et al., 2016; Kuiken et al., 2016; Samuel et al., 2019; Mereu et al., 2021). In an ideal PR-based scheme, it is anticipated that repeatable muscle contraction patterns should be generated for specific limb motion tasks across trials from which feature vector of requisite motor intent is constructed and applied to build machine learning algorithms that decipher the motion intentions of amputees (Li et al., 2010; Asogbon et al., 2020a; Nsugbe et al., 2021a). Besides, various factors could affect the repeatability of muscle activation patterns even for the same limb motion task, which may dampen proper characterization of motion intent and its decoding. One of such factor that has rarely been investigated to date is the EMG-SRD employed to build the machine learning classifier meant to decode the motion task. In an attempt to gain proper insight into how EMG-SRD dynamically impacts the characterization of multiple patterns of elicited limb motion tasks, this study systematically investigated different groups of SRD (1, 5, 10, 15, and 20 s) based on the Within-SRD and Between-SRD strategies using the dataset of eight able-bodied subjects who performed fifteen classes of simple and combined finger gestures. The outcome of the investigation showed that decoding performance across subjects and finger gestures would vary depending on the EMG SRD employed regardless of the feature extraction methods and machine learning classification algorithms utilized.

The Within-SRD has been one of the popularly utilized methods for gesture recognition/classification in myoelectric- PR based systems. Through this method, several studies have reported satisfactory or high classification accuracy for either forearm or finger motion tasks using an average of ≤ 6 s SRD. Unfortunately, the high accuracies reported in all these studies have not translated into robust or intuitive prostheses control schemes that could be widely adopted in clinical and commercial settings. One possible reason may be that the commonly adopted Within-SRD approach in the existing works may not reflect what is obtainable as it relates to practical deployment of the prostheses, which led us to investigate the Between-SRD scenario in the current study. Comparing the performance of the Within-SRD (Figures 3A–C) and Between-SRD (Figures 4A–C), it is obvious that the selection of optimal EMG-SRD should be based on using a more realistic/practical approach (such as the Between-SRD) that will accommodate changes in real-life situations rather than the Within-SRD scenario that has been widely adopted.

Specifically, in the Within-SRD scenario (Figure 3), the average classification accuracies for motion intent decoding were mostly observed to increase with a corresponding increase in EMG signal length for all the features except for a few instances, where the performance either remains approximately the same or declines. Besides, this phenomenon was less obvious when the same set of features were employed on KNN and RF classifiers except for the NTDF feature. Unlike the RMS and TD4 features, the NTDF feature recorded relatively higher average classification accuracies especially for the LDA and KNN classifiers, demonstrating its consistency and stability capabilities. Examining the Between-SRD scenario results (Figures 4A–C), a relatively similar performance trend is seeable with the Within-SRD approach for the features (NTDF, TD4, and RMS) and Classifiers (RF, KNN, LDA) except where LDA + NTDF achieved better performance than KNN + NTDF and RF + NTDF (Figures 3A–C). However, for these two scenarios, this trend is different for the SRD groups suggesting that EMG-SRD will influence the control performance of the prosthesis system in practical applications. In Figures 4A–C, the CA increased as the EMG SRD increased and dropped significantly after 15 s for all the features and classifiers. One potential explanation for the poor performance of 1 s SRD could be that the SRD is too short, and the contained motor information in this short signal recording cannot adequately provide motor information for characterization of the finger gestures. And performance degradation in 20 s SRD could result from fatigue or lack of generalizability of relatively lengthy EMG signal recordings.

In addition to the classification accuracy metric, we investigated the impact of signal recording length on the characterization of the finger motions using the MCC metric and found that the different groups of signal length would result in the varied characterization of the corresponding classes of finger motions (Figures 5A–D). Precisely similar to the result in Figures 4A–C for the Between-SRD scenario, the MCC values increase with a corresponding increase from 1 to 15 s SRD and declined for 20 s SRD. From the plots in Figure 5, overlaps were noticeable for the MCC values of 10 and 15 s SRD, that achieved the best MCC values. Meanwhile, the 1 and 20 s SRD recorded the lowest values. And these results further substantiate the Between-SRD decoding accuracies presented in Figures 4A–C.

To understand how the SRD would impact the characterization of the individual class of finger gesture based on the Between-SRD strategy, we analyzed the fifteen classes of gestures performed across subjects for each group of SRD and observed that signal length would differently influence the classification of the gestures (Figure 6). For all the SRD groups, the thumb-index (TI) finger gesture has the highest accuracy, though with high error bars. From the further examination, the high error bars was because of high-performance variation among the subjects. For instance, participants 5, 6, and 7 recorded higher accuracies for all the SRDs except for 1 and 5 s SRD where participant 5’s performance is like the others. Overall, the decoding accuracies for most finger gestures were higher for 10 and 15 s SRD, with most classes CA overlapping each other. Again, the performance of the SRD groups corroborates our earlier analyses.

Generally, for all the metrics considered in this study for the Between-SRD scenario, the 15 s SRD achieved the highest performance, followed by 10 s for the combination of RF + NTDF. Nevertheless, it is noteworthy to state that the performance difference in these two SRD groups (10 and 15 s) is not statistically significant (*p* > 0.05), almost the same or slightly different (<1–2% for CA and MCC metrics), and also increased SRDs would lead to increase computational cost. Therefore, we would suggest a signal recording length of greater than 5 s but less than or equal to 10 s (>5 and ≤ 10 s) as being potential considering the fact that the lengthier the EMG recordings the more processing time it may require to build the classifier. Findings from our study suggest that the may be a safe zone in terms of the SRD.

Finally, the main strength of this study is that it provided a proper insight into the impact of SRD on EMG classification accuracy using three different time domain features and machine learning classification algorithms and how to select the optimal SRD that would be robust in practical situations. To the authors’ knowledge, this has not been previously investigated. Moreover, it is essential to mention the drawbacks of this study. Firstly, the dataset was acquired from only healthy subjects with three trials for each class of finger gestures. Secondly, the presented experimental results were based on offline analysis. In our future work, we hope to recruit more healthy subjects and amputees from which a wider range of gestures (including finger, forearm, and wrist movements) will be obtained to further validate our hypothesis. It also worth mentioning that our future investigations shall be done in an online setting other than the offline analysis carried out in the current study, employing real-time evaluation metrics to further validate our hypothesis.

## 5. Conclusion

Pattern recognition-based electromyogram control method for prostheses has been highlighted and demonstrated as a potential control strategy that can aid the realization of multiple degrees of freedom prosthetic functions in a dexterous manner. Besides, an important aspect of the framework that has rarely been investigated is the myoelectric SRD upon which multiple classes of limb motion tasks are characterized. Thus, this study systematically investigated the impact of varying EMG-SRD on the characterization of motor intents associated with multiple classes of finger gestures. The investigation involved characterizing fifteen classes of finger gestures performed by eight normally limb subjects under varying lengths of EMG-SRD (1, 5, 10, 15, and 20 s). Thereafter, each group of recordings was pre-processed followed by the extraction of different feature sets, and machine learning classification algorithms were employed for decoding the corresponding gestures based on two strategies namely Between-SRD and Within-SRD scenarios. Comparison between these scenarios revealed that EMG-SRD would influence the performance of motion intent decoding. In the experimental results for Between-SRD scenario, SRDs of 10 and 15 s yielded reasonably decent performance compared to other SRDs in terms of movement intent decoding across finger gestures, subjects, feature sets, and classifiers. Considering the increased computation complexity that comes with increased SRD and the fact that no significant/substantial improvement was seen in the performance of 10 and 15 s SRD, the study will recommend a longer SRD (>5 and ≤ 10 s) depending on the research objective. The optimal SRD was determined based on the Between-SRD approach because it is more realistic/practicable compared to the Within-SRD scenario. More importantly, determining the optimal signal length is crucial to adequately characterize multiple classes of targeted limb motions in the context of EMG-PR-based control for multifunctional prostheses. In our future work, further investigations will be conducted to validate the findings of this study.

## Data availability statement

Publicly available datasets were analyzed in this study. This data can be found here: https://www.rami-khushaba.com/biosignals-repository.

## Ethics statement

The studies involving human participants were reviewed and approved by we used a public dataset whose URL is: https://www.rami-khushaba.com/biosignals-repository. The patients/participants provided their written informed consent to participate in this study.

## Author contributions

MA, OS, and GL: conceptualization, funding acquisition, and writing – review and editing. MA and OS: data curation, writing – original draft, and methodology. OS and GL: supervision. YL and DM: further data analysis and result interpretation. EN, FK, and SC: validation. MA, OS, SC, EN, and FK: visualization. All authors contributed to the article and approved the submitted version.
